# Development of Respiratory Syncytial Virus Vaccine Candidates for the Elderly

**DOI:** 10.3390/v15061305

**Published:** 2023-05-31

**Authors:** Jorge C. G. Blanco, Lori M. Cullen, Arash Kamali, Fatouomata Y. D. Sylla, Marina S. Boukhvalova, Trudy G. Morrison

**Affiliations:** 1 Sigmovir Biosystems Inc., Rockville, MD 20850, USA; 2 Department of Microbiology and Physiological Systems, University of Massachusetts Chan Medical School, Worcester, MA 01655, USA

**Keywords:** respiratory syncytial virus, vaccine, virus-like particle, immunity

## Abstract

Respiratory syncytial virus (RSV) is a significant threat to elderly populations and repeated infections that occur throughout life are poorly protective. To assess the role of prior RSV infections as well as elderly immune senescence on vaccine efficacy, we compared immune responses after virus-like particle (VLP) immunization of elderly cotton rats and young cotton rats, both previously RSV infected, in order to mimic the human population. We show that immunization of RSV-experienced young or elderly animals resulted in the same levels of anti-pre-F IgG, anti-G IgG, neutralizing antibody titers, and protection from challenge indicating that the delivery of F and G proteins in a VLP is equally effective in activation of protective responses in both elderly and young populations. Our results suggest that F and G protein-containing VLPs induce anti-RSV memory established in prior RSV infections equally well in both young and elderly animals and thus can be an effective vaccine for the elderly.

## 1. Introduction

Respiratory syncytial virus (RSV) is an important cause of acute lower respiratory infections that are particularly serious in infants, young children, and the elderly. It has been estimated that there are, worldwide, 30 million infections/year of infants [1,2,3], ten percent of which require hospitalization [2,4]. RSV infections of the elderly rival influenza in severity of disease [2,3,5,6]. Vaccines targeting different at-risk populations may require different formulations or different protocols for administration. Effective, durable vaccines focused on the elderly need to consider immune senescence in this population [7,8,9].

The importance of developing vaccines designed for use in elderly populations is clear given that the virus results in an estimated 11,000 to 17,000 deaths per year in the US and ten times that number of RSV-associated hospitalizations [2,3,10]. Furthermore, elderly populations are projected to significantly increase in the next few decades. It has been forecast that the worldwide population over age 60 will reach 1.6–2.1 billion, more than 20% of the population, by 2050 [11] (World Population Aging Report 2015, United Nations). In some developed countries, this percentage is already at 20–25% (World Population Aging Report 2015, United Nations). Such an expansion in the elderly population will pose a greatly increased public health burden and RSV infections will contribute to that burden without an effective vaccine.

It is well recognized that the elderly have reduced immune responses to many vaccines compared to young adults [7] due to a phenomenon termed immune aging or immunological senescence (for example [7,12,13]). The nature of this senescence is incompletely understood, but in the case of RSV, immunization of the elderly must consider also that most, if not all, of the human population, has been infected by age two, and individuals may be re-infected multiple times during life [1]. There are two antigenic groups of RSV, A and B, and there are multiple genotypes within both groups based on the variable region of the G protein [14]. However, as reviewed by Pangesti [14], the evidence does not clearly support the idea that reinfections are always due to different virus subtypes or genotypes. Rather there is evidence that individuals can be re-infected with either the same genotype or a different genotype or subtype. Thus, the appearance of different antigenic variants may not fully account for repeated infections throughout life. Furthermore, a successful RSV vaccine candidate must stimulate high titers of neutralizing antibodies in the context of any preexisting but poorly protective immunity while overcoming immune senescence. Studies of the efficacy of RSV vaccine candidates in RSV naïve young or RSV naïve elderly animal models may not accurately reflect protective responses in humans.

To address the role of prior RSV infection as well as age on vaccine efficacy, we compared responses induced by immunization with virus-like particle (VLP) vaccine candidates in young and old animals both of which had been previously infected with RSV at an early age [15,16]. We show that VLP immunization of these RSV-experienced elderly as well as young animals resulted in the same levels of anti-pre-F IgG, anti-G IgG, neutralizing antibody titers, and levels of protection from RSV challenge, indicating that the VLPs are equally effective in activation of protective responses in both populations. Our results are consistent with the idea that natural immunity to RSV, acquired by previous infection at a young age, produces a memory response that can be efficiently activated in both young and elderly cotton rats by VLP immunization.

## 2. Materials and Methods

### 2.1. Cells, Virus, Plasmids

ELL-0 and HEp-2 cells, obtained from the American Type Culture Collection (ATCC, Manassas, VA, USA) were grown in DMEM (Invitrogen, Carlsbad, CA, USA) supplemented with penicillin, streptomycin (Invitrogen, Carlsbad, CA, USA), and 10% fetal calf serum (Invitrogen, Carlsbad, CA, USA). Expi293F, obtained from Invitrogen, (Carlsbad, CA, USA) was grown in Expi293 media (Invitrogen, Carlsbad, CA, USA). The prototype of the Long strain of RSV was obtained from the American Type Culture Collection (ATCC VR-26, Manassas, VA, USA). RSV, A2 strain, was obtained from Dr. Robert Finberg. The virus was propagated in HEp-2 cells and serially plaque-purified to reduce defective-interfering particles. A single pool of RSV A/Long virus containing 10^7.6^ PFU/mL was used for all experiments. To adjust the dose, stock aliquots were diluted with PBS for intranasal (IN) infections. Viral titers in the lungs of RSV naïve animals were determined as previously described [17].

### 2.2. Preparation, Characterization, and Validation of VLP Stocks

VLPs used as immunogens were based on the core proteins of Newcastle disease virus (NDV) M and NP proteins and contained the RSV F and G glycoproteins [18,19,20]. The RSV proteins were assembled into the VLPs as chimera proteins with the sequences of the ectodomain of RSV F and G glycoproteins (sequences from RSV subtype A2) fused to the transmembrane and cytoplasmic domains of the NDV F and HN proteins, respectively, to generate F/F and H/G chimera proteins [19]. Two different VLPs were prepared, each containing the same H/G chimera protein but with a different mutant F chimera protein. One VLP contained the DS-Cav1 pre-fusion F protein [21], while another VLP contained the mutant UC-3 F [21], a pre-fusion F protein with the cleavage site and intervening p27 sequences replaced with a seven-amino acid GS rich linker sequence as well as three point mutations, N67I, S215P, and D486N, similar to the SC-TM F protein described by Krarup et al. [22]. The two pre-fusion F proteins also contained the foldon sequence inserted between the RSV F protein ectodomain and the NDV F protein transmembrane domain to stabilize further the pre-fusion conformation [20,23].

The VLPs were prepared by transfecting avian cells (ELL-0 cells, ATCC), growing in T-150 flasks) with cDNAs encoding the NDV M and NP proteins, the H/G protein chimera, and one of the mutant F chimera proteins (DS Cav1 F/F or UC-3 F/F) inserted into the pCAGGS vector [19]. VLPs released into the cell supernatant were purified as previously described [24] and the F and G protein content of purified VLPs was were quantified by Western blots and VLP stocks were adjusted for equivalent levels of F protein [16,21]. The pre-fusion conformation of the F proteins was validated by binding of mAb specific for the pre-fusion F protein, mAb D25 and mAb AM14, as previously described [16,21,25].

### 2.3. Preparation of Soluble F and G Proteins

Expi293F cells were transfected with cDNAs encoding the soluble DS-Cav1 pre-F protein or the soluble UC-3 pre-F protein or the soluble G protein. At six days post-transfection, total cell supernatants were collected, cell debris removed by centrifugation, and the soluble polypeptides were purified on columns using the His tag and then the streptavidin tag as previously described [16,26]. Purified soluble DS-Cav1 pre-F protein and soluble UC-3 pre-F protein were validated by binding to pre-fusion specific mAbs AM14 and D25 [21].

### 2.4. Quantification of Soluble and VLP-Associated F and G Proteins

Determinations of amounts of RSV F protein and G protein in soluble F protein or soluble G protein preparations or in VLPs were accomplished by Western blots using anti-HR2 antibody or anti-G protein antibody for detection and comparing the signals obtained with a standard curve of purified F proteins as previously described by Cullen et al. [16,21].

### 2.5. Preparation of RSV, RSV Plaque Assays, and Antibody Neutralization

RSV was propagated in HEp-2 cells (ATCC CCL-23), and RSV plaque assays were accomplished on HEp-2 cells as previously described [16,26]. RSV NAb titers were measured by 60% plaque reduction assay. Briefly, four- or six-fold dilutions of heat-inactivated serum samples were incubated with 50–100 PFU of RSV A/Long and then inoculated onto HEp-2 cells. After 4 days of incubation under 0.75% methylcellulose overlay, the overlay was removed, and the cells were fixed and stained with crystal violet. Plaques were quantified and reciprocal NAb titers were determined as previously described (the limit of detection of this assay is 4.32 Log_2_) [17].

### 2.6. Antibodies

Antibodies used for Western blots were anti-RSV HR2 raised in rabbits using as antigen the RSV F HR2 domain, as previously described [19]. Anti-RSV G protein antibody is a polyclonal antibody raised against a peptide containing G protein amino acids 180–198 (ThermoFisher, Waltham, MA, USA). Monoclonal antibodies specific to F protein and used to validate soluble and VLP-associated F protein were D25, palivizumab, AM14, and motavizumab and were obtained from B. Graham, J. Blanco, and J. McLellan, respectively. G protein-specific mAb used were 131-2G (Millipore Sigma MAB858-2, Burlington, MA, USA) and mAb1187 (from J. Beeler). Monoclonal antibodies MPE8 and 5C4 were humanized versions of anti-F protein-specific murine antibodies. Secondary antibodies against goat, mouse, human, and rabbit IgG were purchased from Sigma.

### 2.7. Animals

**Figure 1:**
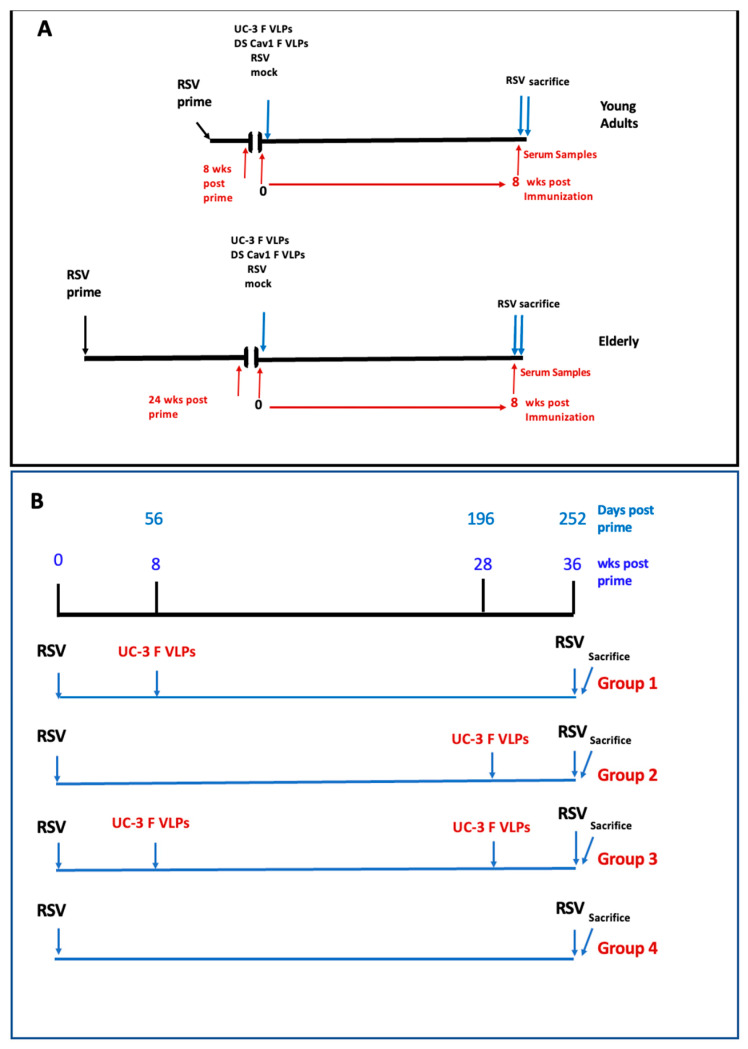
Experimental Design. (**A**): Protocol 1: Impact of age on immune responses: eight groups of ten 4–6-week-old CR were infected, (IN), with RSV (RSV primed). After 8 weeks, four groups were immunized with UC-3 F/F+H/G VLPs, or DS Cav1 F/F +H/G VLPs, or infected a second time with RSV (IN), or mock immunized (young). After another 8 weeks, these animals were challenged with RSV and sacrificed four days later. A second cohort of four groups of ten animals were similarly immunized at 24 weeks after the RSV prime (elderly). Eight weeks after immunization, these animals were challenged with RSV, (IN), and sacrificed four days later. (**B**): Protocol 2: Impact of two immunizations on immune responses: forty 4–6-week old CR were divided into four groups of ten animals. All animals were infected with RSV (RSV prime, day 0). Group 1 CR were immunized with UC-3 F/F+H/G VLPs on day 56 and mock immunized on day 196. Group 2 CR were mock immunized on day 56 and immunized with UC-3 F/F+H/G VLPs on day 196. Group 3 CR were immunized with UC-3 F/F+H/G VLPs on both days 56 and 196. Group 4 CR were mock immunized on day 56 and day 196. All animals were challenged with RSV on day 252 and sacrificed on day 256.

*Sigmodon hispidus* cotton rats were from the inbred colony maintained at Sigmovir Biosystems, Inc. (Rockville, MD, USA). Four- to six-week-old cotton rats from both sexes were recruited for these studies and randomly tagged and separated into the indicted groups (Figure 1). Animals were pre-bled before inclusion in the study to rule out the possibility of pre-existing antibodies against RSV. The colony was monitored for antibodies to paramyxoviruses and rodent viruses and no such antibodies were found. All studies were conducted under applicable laws and guidelines and after approval from the Sigmovir Biosystems, Inc. Institutional Animal Care and Use Committee (IACUC). The experiments in these studies follow IACUC protocol 15 approved and valid until 31 October 2025. Animals were housed in large polycarbonate cages and fed a standard diet of rodent chow and water ad libitum.

### 2.8. Lung Histopathology

Lungs were dissected and inflated with 10% neutral buffered formalin to their normal volume, and then immersed in the same fixative solution. Lungs were then embedded in paraffin, sectioned, and stained with hematoxylin and eosin (H&E). An average pathology score was determined for each group based on four parameters of pulmonary inflammation: peribronchiolitis (inflammatory cell infiltration around the bronchioles), perivasculitis (inflammatory cell infiltration around the small blood vessels), interstitial pneumonia (inflammatory cell infiltration and thickening of alveolar walls), and alveolitis (cells within the alveolar spaces), illustrated in Figures S1 and S2. Slides were scored blindly on a 0–4 severity scale.

### 2.9. RSV Gene Expression by Real-Time PCR

Total RNA was extracted from homogenized lung tissue using the RNeasy purification kit (Qiagen, Germantown, MD, USA). One μg of total RNA was used to prepare cDNA in a volume of 20 mL (QuantiTect Reverse Transcription Kit from Qiagen). cDNA was diluted to 10 μg/mL and 3 μL was used for each 25 μL real-time PCR reaction (QuantiFast SYBR Green PCR Kit from Qiagen, Germantown, MD, USA) with final primer concentrations of 0.5 μm. Primer sequences for RSV NS1 quantification are 5′-CACAACAATGCCAGTGCTACAA; 5′-TTA GAC CAT TAG GTT GAG AGC AAT GT; for direct and reverse set, respectively. Reactions were set up in 96-well plates and amplifications were performed on a Bio-Rad iCycler (MyiQ Single Color Real Time PCR Detection System, Software Version 1.0, Hercules, CA, USA). 2^−ΔΔC*t*^ method was used to calculate relative gene expression after normalization to actin as the chosen housekeeping gene as described [27].

### 2.10. ELISA

For the determination of anti-pre-F protein or anti-G protein IgG antibody titers, wells of microtiter plates (ThermoFisher/Costar, Waltham, MA, USA) were coated with either purified soluble DS-Cav1 F protein, soluble UC-3 F protein, or soluble G protein (30 ng/well) and incubated overnight at 4 °C, then blocked with 2% BSA for 16 h at 4 °C. Different dilutions of sera, in PBS-2% BSA and 0.05% Tween, were added to each well and incubated for 2 h at room temperature. Wells were then washed with PBS, incubated with chicken anti-cotton rat IgG antibody (Abnova PAB29753) coupled to HRP, and incubated for 1.5 h at room temperature. Bound HRP was detected using TMB (3,3′5,5′-tetramethylbenzidin, ThermoFisher34028), and the reaction was stopped with 2N sulfuric acid. The color was read in SpectraMax Plus Plate Reader (Molecular Devices, San Jose, CA, USA) using SoftMax Pro software. Amounts of IgG (ng/mL) in each dilution were calculated using a standard curve generated using defined amounts of purified cotton rat IgG.

### 2.11. Experimental Design

Protocol one: impact of age on immune responses. Eight groups of ten 4–6-week-old cotton rats (CR) were infected (intranasal infection, IN), with RSV A/Long (RSV primed) using a dose of 10^5^ PFU/animal in 50 μL. After 8 weeks, four groups (young) were immunized by intramuscular injection (IM) with UC-3 F/F+H/G VLPs, or DS Cav1 F/F+H/G VLPs (doses of 100 μg total VLP protein/animal containing 20 μg F protein), or infected a second time with RSV by intranasal administration (IN) using a dose of 10^5^ PFU/animal in 50 μL, or mock immunized with 50 μL TNE buffer (50 mM Tris-HCl, pH 7.4, 150 mM NaCl, 5 mM EDTA). After 8 weeks, these animals were challenged with RSV A/Long (10^5^ PFU/animal) and sacrificed four days later (Figure 1A).

The second cohort of four groups of ten animals were similarly immunized at 24 weeks after the RSV prime (elderly). Eight weeks after immunization, these animals were similarly challenged with RSV, (IN), and sacrificed four days later (Figure 1A, bottom).

Protocol 2: Impact of Two Immunizations on Immune Responses. Forty 4–6-week-old CR were infected IN with RSV A/Long using a dose of 10^5^ PFU/animal in 50 μL (RSV prime, day 0). These animals were then divided into four groups of ten animals. Group 1 CR was immunized, IM, with UC-3 F/F+H/G VLPs on day 56 and mock immunized on day 196 with 50 μL TNE buffer. Group 2 CR was mock immunized with TNE buffer on day 56 and immunized, IM, with UC-3 F/F+H/G VLPs (100 μg VLP protein, 20 μg F protein) on day 196. Group 3 CR were immunized, IM, with UC-3 F/F+H/G VLPs (per animal 100 μg total VLP protein containing 20 μg F protein in 50 μL) on day 56 and again on day 196. Group 4 CR was mock immunized with TNE buffer on day 56 and day 196. All animals were challenged with RSV A/Long (10^5^ PFU/animal) on day 252 and sacrificed on day 256.

### 2.12. Statistical Analysis

Statistical analyses used ANOVA (followed by a *post hoc* Tukey HSD or Sidák multiple comparison test) or Student’s-*t* test of data (indicated in the figure legends) all of which were accomplished using Graph Pad Prism 9 software.

## 3. Results

### 3.1. Impact of Age of Immunization on Immune Responses

Cotton rats (CR) are a widely accepted animal model for RSV studies of vaccine candidates [28,29] and were used here to ask if the age of immunization, young vs. old, impacted immune responses to VLP immunization at 8 weeks after immunization (Figure 1A). All animals in Protocol 1 were first primed with RSV IN (10^5^ PFU IN in 50 μL) to resemble natural conditions in the elderly. Groups of animals were immunized 56 days after RSV priming (immunization while young) while other groups of animals were immunized 168 days after RSV priming (immunization while elderly). Four groups of young and four groups of elderly animals received either UC-3 F/F+H/G VLPs, DS-Cav-1 F/F+H/G VLPs, a second RSV infection, or mock immunizations (Figure 1A). All animals were challenged with RSV (IN) at 8 weeks after immunization and sacrificed four days later. Serum samples in all animals were acquired at the time of immunization (day 56 or day 168) and at 8 weeks after immunization, just before the RSV challenge, for the assessment of antibody and serum neutralization titers (young animals at day 112 and elderly animals at day 224) (Figure 1A).

#### 3.1.1. Total Anti-Pre-F and Anti-G Protein IgG Titers 8 Weeks after VLP Immunization

We first asked if there was a difference in total anti-pre-F IgG or total anti-G IgG 8 weeks after immunization of young vs. elderly CR.

**Figure 2:**
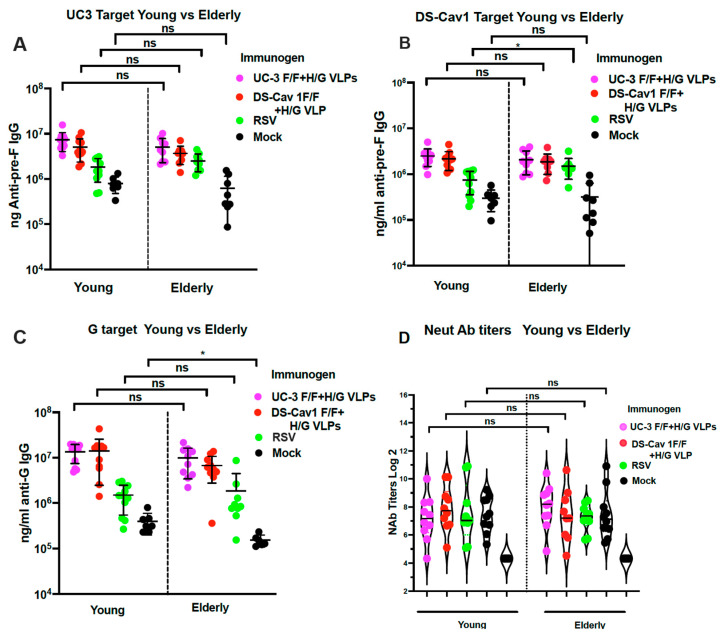
Immune responses of animals in Protocol 1. The ng/mL of anti-pre-F IgG and anti-G IgG were determined in sera collected from young and elderly CR at 8 weeks after immunization. Levels of anti-pre-F IgG were measured by ELISA using soluble UC-3 F protein (**A**) or soluble DS Cav-1 F protein (**B**) as targets. Levels of anti-G IgG were measured by ELISA using soluble G protein as the target (**C**). Data from each animal are shown with the mean and standard deviation of titers indicated. (**D**) shows neutralizing antibody titers (mean and standard deviation) in each animal in each group. Statistical analysis of differences between young and elderly animals receiving the same immunogen was determined by Student’s *t*-test. * *p* < 0.02. ns: non-significant.

The total anti-pre-F IgG antibodies and anti-G IgG antibodies were measured by ELISA using as targets soluble UC-3 pre-F protein, soluble DS Cav1 pre-F protein, or soluble G protein (Figure 2A–C, respectively). The two different pre-fusion F targets were used because we previously detected differences in immune responses using the two targets [30].

Comparison of levels of anti-pre-F IgG and anti-G IgG antibodies in young and elderly CR showed no statistically significant differences between the two age groups indicating that elderly CR were just as competent for VLP activation of anti-pre-F and anti-G IgG as young animals. Importantly, a second RSV infection induced significantly lower levels of antibodies than the VLP vaccination in young and elderly animals, as we have previously reported [15,16], whereas mock immunized animals (RSV-primed only) showed even lower titers of antibodies in both groups.

#### 3.1.2. Total Neutralizing Antibody Titers 8 Weeks after VLP Immunization

We next asked if titers of RSV-neutralizing antibodies (NAb) were impacted by the age of immunization. NAb titers were measured in the sera acquired from the two populations of animals (Figure 2D) just prior to the RSV challenge. There were no statistically significant differences in NAb titers at 8 weeks after vaccination of young vs. elderly animals, again suggesting that the elderly were able to respond to immunization as effectively as the young animals.

These combined results are consistent with the idea that RSV infection in young animals can induce memory responses that can be just as effectively recalled by VLP immunization in elderly animals as they are in young animals.

### 3.2. Impact of One vs. Two Immunizations at Different Ages

#### 3.2.1. Protocol 2

We next asked if immunization at a young age followed by a boost at old age increased protective immune responses in the elderly (Protocol 2, Figure 1B). Important controls for this question were a group of CR immunized only at a young age, (group 1), and a second group of CR immunized only at an old age (group 2). The experimental group was immunized at a young age and then boosted at an old age (group 3, Figure 1B).

**Figure 3:**
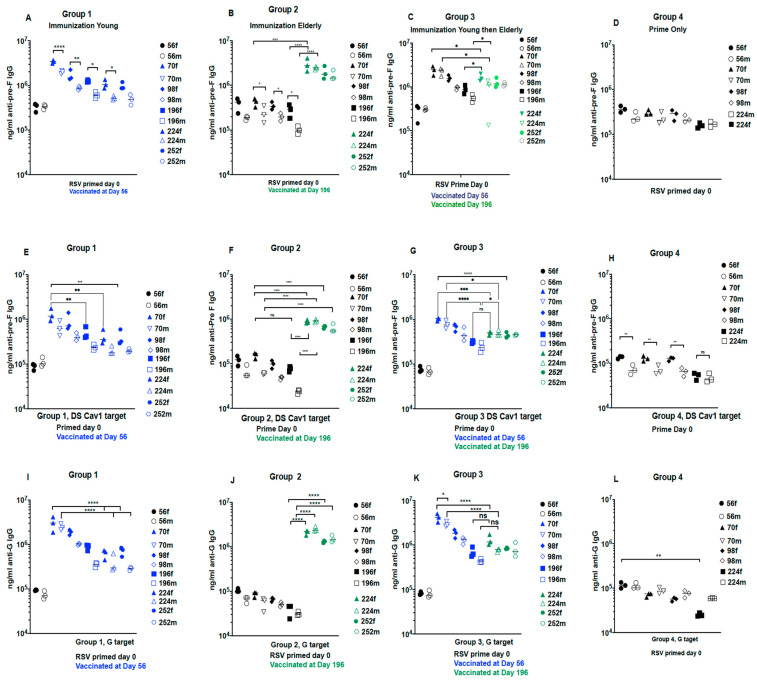
Anti-IgG titers in animals in protocol 2. The sera from females and from males in each group at each time point were pooled separately. The ng/mL of anti-pre-F IgG in the sera pools from all four groups of males and females at each time point was measured by ELISA with soluble UC-3 F protein as target (**A**–**D**) or soluble DS-Cav1 F as target (**E**–**H**). The ng/mL of anti-G IgG was measured by ELISA using soluble G protein as the target (**I**–**L**). Each serum pool was assessed three times and the results of all three assays are shown with the averages indicated by a black line. Closed symbols, females; open symbols, males. Statistical analyses of differences between groups and sex differences were accomplished by one-way ANOVA, with Sidak’s multiple comparisons test. * *p* < 0.02; ** *p* < 0.005; *** *p* < 0.0005; **** *p* < 0.0001. ns: non-significant.

For this experiment (Figure 1B), four groups of 10 CR each, 4–6 weeks of age, were infected (IN) with RSV (day 0, priming). Group 1 received a single dose of UC-3 F/F+H/G VLPs on day 56 after RSV priming. Group 2, instead, received a single dose of UC-3 F/F+H/G VLPs on day 196 after RSV priming (at 8 months of age), while group 3 received two doses of UC-3 F/F+H/G VLPs, one on day 56 and one on day 196. The animals in group 4 were RSV-primed but mock-immunized. Another control group of ten animals was unprimed and uninfected. All animals were challenged with RSV, IN, on day 252 and sacrificed 4 days later. Sera were acquired throughout the protocol as indicated in Figure 3.

#### 3.2.2. Total Anti-Pre-F and Anti-G Antibodies in Sera with Time after Immunization

We first asked if two immunizations induced superior total anti-pre-F and anti-G IgG compared to a single immunization. Figure 3 shows the time courses of total anti-pre-F and G IgG in each CR group as measured by ELISA. Sera from each group, at each time point, were pooled for ELISA, keeping pools of sera from males and females separate. Data from individual animals in all groups immunized with UC-3 F/F+H/G VLPs are shown in Figure S1.

Figure 3C,G,K, show that a second immunization in elderly (group 3) CR did significantly boost levels of anti-Pre-F and anti-G IgG over that detected just prior the second immunization at day 196.

The mock immunized CR (group 4 animal, Figure 3D,H,L), showed that the low levels of antibody induced by the RSV prime declined approximately 0.5 logs with time.

All panels show that, in most groups at most time points, responses in female CR were higher than responses in male animals with many of the differences statistically significant.

**Figure 4:**
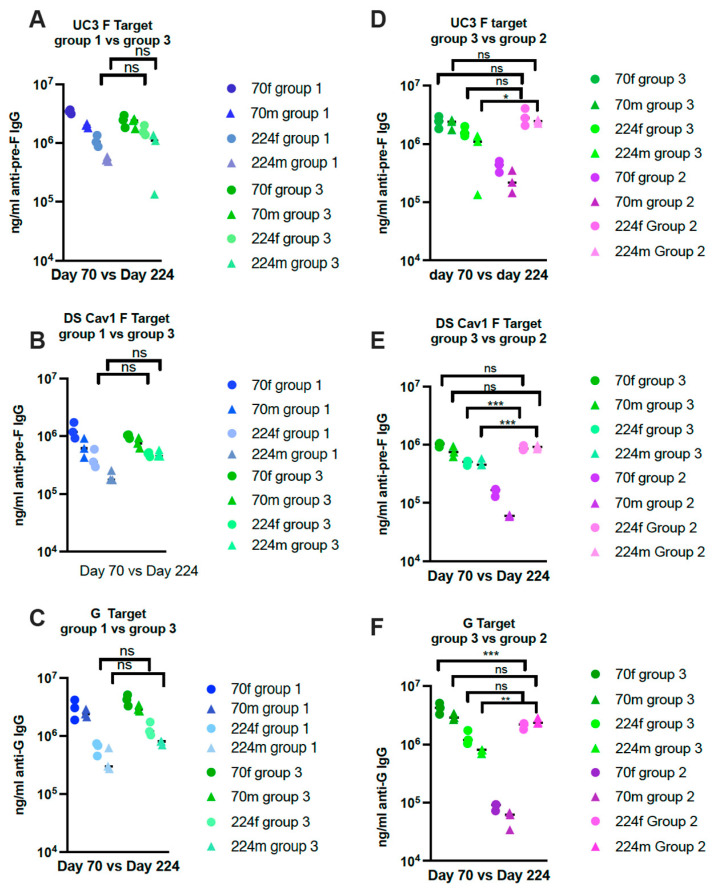
Comparisons of IgG titers between key groups in Protocol 2. Comparisons of titers of anti-pre-F IgG or anti-G IgG in groups 1 and 3 on days 70 and 224 (two weeks after immunization) are shown in (**A**–**C**) using soluble UC-3 F (**A**) or soluble DS-Cav1 F (**B**) or soluble G protein (**C**). (**D**–**F**) show comparisons of IgG ELISA titers at day 70 vs. 224 in groups 2 and 3 animals using soluble UC-3 F (**D**) or soluble DS-Cav1 F (**E**) or soluble G protein (**F**). The statistical significance between key time points was determined by one-way ANOVA with a Tukey multiple comparison test. Circles: females; triangles: males.* *p* < 0.02; ** *p* < 0.005; *** *p* < 0.0005; ns: non-significant.

A direct comparison of levels of IgG at key points in different groups is shown in Figure 4. Importantly, the levels on day 224 in group 3 animals after the second immunization were not statistically different from those in group 1 (Figure 4A–C) and group 2 on day 224. (Figure 4D–F). Although a second immunization increases titers in the same group of animals, the comparisons of levels across different groups suggest that a second immunization in the elderly may have minimal added benefits to a primary immunization in the young or the elderly.

#### 3.2.3. Impact of Times of Immunization on Neutralization Titers in Sera

**Figure 5:**
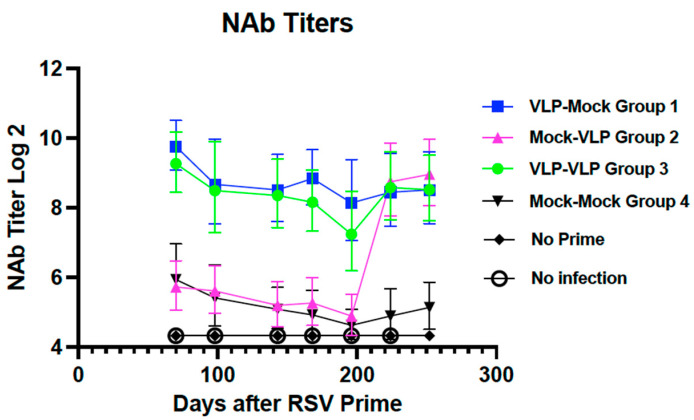
Neutralization titers in pooled sera from Protocol 2. Using pools of sera generated for measures of total IgG as described in Legend to Figure 3 but with sera from females and males combined, the neutralization titers with time were determined in a classical plaque reduction assay. Means and standard deviations are shown.

We next asked how levels of total anti-pre-F and anti-G IgG titers correlated with neutralizing antibody titers (NAb). Figure 5 shows NAb titers of pools of all sera at each time point after immunization. Group 1 and Group 3 sera had similar average NAb titers at all time points. There was no significant increase in titers of group 3 animals after a second immunization on day 196. Surprisingly, there was no decrease with time in titers in group 1 animals, which was in contrast to total IgG levels with time after immunization at day 56.

There was a dramatic increase in NAb titers in group 2 sera after a single immunization at an old age, on day 196, to titers equivalent to the maximal titers reached by groups 1 and 3. Thus, with the exception of late times in group 1 animals, NAb titers generally tracked with total anti-F or anti-G IgG.

**Figure 6:**
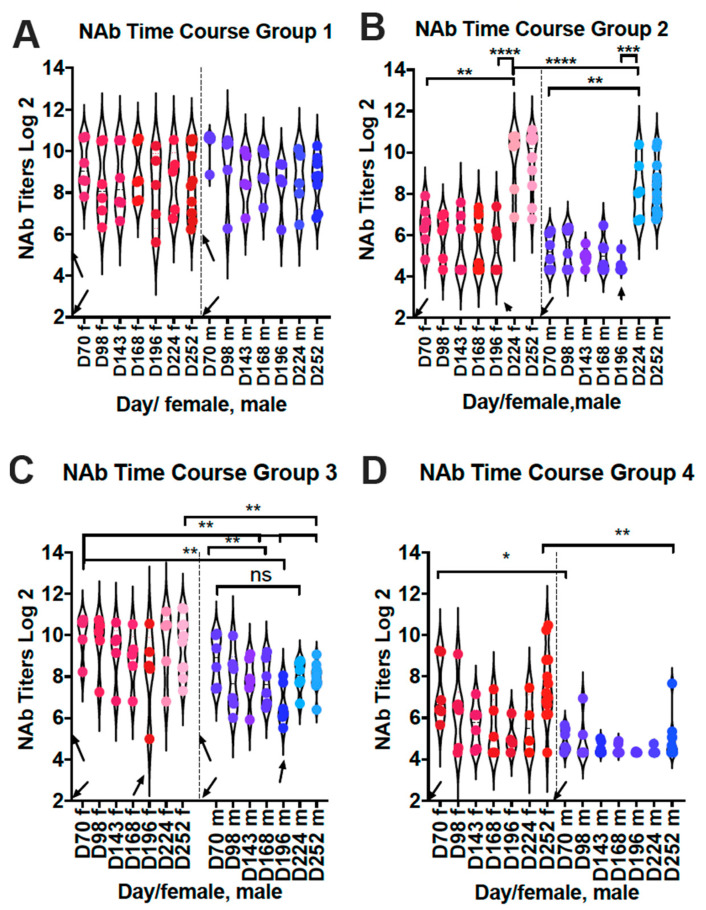
Neutralization titers in individual animals from Protocol 2. NAb titers, determined in a plaque reduction assay, were measured in each animal at each time point. Results from female (red symbols) and male animals (blue symbols) are presented separately. Each panel (**A**–**D**) represents the results of the different vaccination groups (groups 1, 2, 3, and 4, respectively). Downward arrows indicate time of RSV priming. Upward arrows indicate times of immunization. D = day; f = female; m = male. Statistical significance between key points was measured by one-way ANOVA with Sidak’s multiple comparisons test: * *p* < 0.02; ** *p* < 0.005; *** *p* < 0.0005; **** *p* < 0.0001; ns: non-significant.

Figure 6A–D compare the NAb titers in individual animals at each time point in each group, and differentiate between sexes. In group 1 control animals, there was no statistically significant difference in NAb titers throughout the time course (Figure 6A) in contrast to total anti-pre-F IgG. That is, the NAb titers in animals in group 1 do not significantly decline as do total anti-pre-F and anti G antibodies. Similarly, the NAb titers in group 3 animals just prior to the second immunization did not drop as dramatically as the total IgG levels. As a result, the boost in NAb titers after the second immunization were less dramatic. These combined results suggest that different populations of antibodies have different stabilities. However, NAb titers in group 2 and group 3 animals do generally track with the levels of total anti-pre-F and anti-G antibodies.

Our data also indicate that, as previously reported by others [31], there are differences in antibody responses in males and females, both total IgG and NAb titers. In group 1 animals, there were no statistically significant differences in NAb titers, although male titers tended lower. However, in group 2 animals, there was a significant difference in titers between males and females with female titers higher than in males. In group 3 animals, NAb titers in males after the second immunization were significantly lower than those in females.

NAb titers in group 4 animals plot durability of the titers after the RSV prime without additional immunization (Figure 6D). In contrast to VLP stimulated antibodies, there was a decline in the NAb titers with time, in both females and males. These combined results indicate that there are significant differences in NAb titers in females and males with the female titers higher.

#### 3.2.4. Impact of Age on Protection from RSV Replication by VLP Immunization

**Figure 7:**
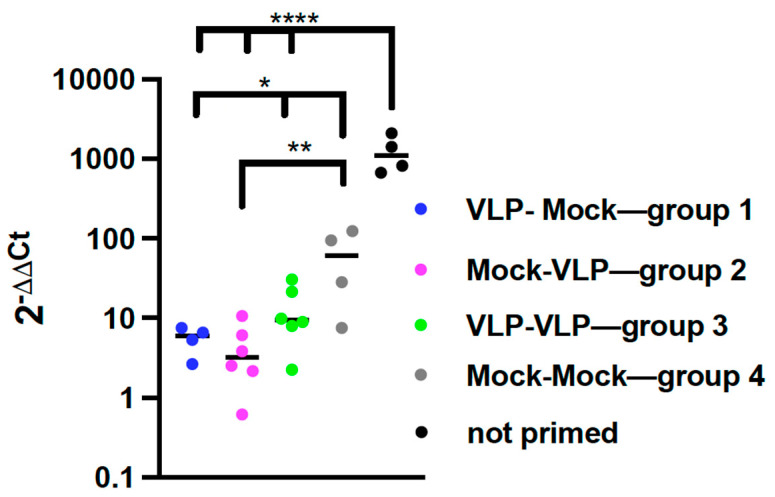
Protection of Protocol 2 animals from RSV challenge. On day 252, all animals in Protocol 2 were challenged with RSV and then sacrificed on day 256. The levels of RSV NS-1 transcript in the lungs of the animals were measured by qRT PCR. Results from individual animals are shown. Statistical analysis of differences between results with group 4 animals compared to results with groups 1, 2, and 3 was accomplished by one-way ANOVA with Dunnett’s multiple comparisons test. Separately, analysis of differences in results from groups 1–3 animals with those of uninfected unvaccinated animals with was similarly accomplished by one-way ANOVA with Dunnett’s multiple comparisons test. * *p* < 0.05 or 0.02; ** *p* < 0.005; **** *p* < 0.0001.

We next asked if two immunizations resulted in increased protection from RSV challenge compared to a single immunization at young or at old age. Cotton rats previously infected with RSV develop lifelong protective immunity. However, in RSV primed CR, abortive RSV replication occurs during a second infection, as indicated by expression of RSV mRNA, and correlates with the lung pathology that is evident after the second RSV challenge [32]. Thus, to measure protection from RSV challenge afforded by VLP immunization in these experiments, the levels of RSV NS-1 RNA transcripts in lungs of groups 1, 2, and 3 animals four days after RSV challenge were quantified by qRT-PCR and compared to levels in the RSV primed, mock immunized animals (group 4). The levels of RSV NS-1 RNA transcripts in the lungs of infected but unprimed, unimmunized animals were also included as a control. Figure 7 shows that groups 1, 2, and 3 animals expressed significantly less NS-1 RNA transcripts in lungs compared to the RSV-primed, unimmunized animals demonstrating that VLP immunization activates protective immune responses. There were no statistically significant differences in levels of NS-1 RNA transcripts in groups 1, 2, or 3 animals although the NS-1 expression in group 2 animals trended lower than that of groups 1 and 3. These results show that a single VLP immunization of elderly animals is just as effective in providing enhanced protection from RSV challenges as immunization once in young animals, or twice, at young and old ages.

#### 3.2.5. Assessment of Lung Pathology after RSV Challenge of CR Immunized Once or Twice with VLPs

**Figure 8:**
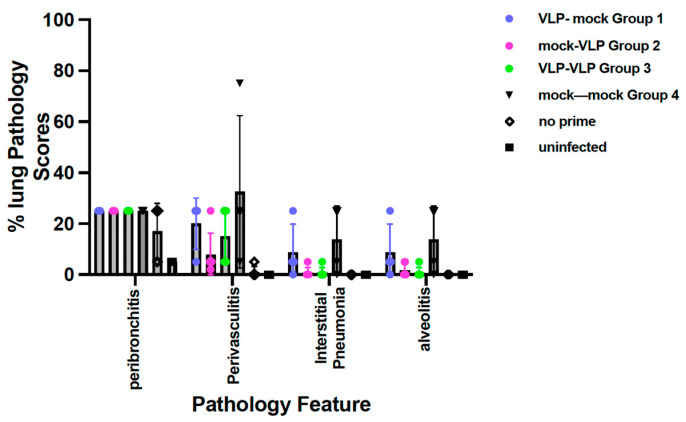
Pathology scores in animals in Protocol 2 after RSV challenge. The means and standard deviations of scores for peribronchiolitis, perivasculitis, interstitial pneumonia, and alveolitis for all animals in each group are shown.

We asked if immunization of elderly CR enhanced pathology after an RSV challenge compared to immunization in young CR or twice in CR (Figure 8). Peribronchiolitis was the most significant pathology in all immunized animals but there were no differences in scores between groups of immunized animals and RSV primed, mock immunized animals. The pathology scores in other categories were lower in all four groups of CR. There was a trend for less perivasculitis, interstitial pneumonia, and alveolitis in group 2 and group 3 animals compared to group 1 animals.

Representative images of lungs from each group are shown in Figures S2 and S3. While the differences were not statistically significant, the consistently higher scores in group 1 animals, immunized only as young adults, may indicate that immunization later in life results in less pathology upon RSV challenge.

## 4. Discussion

The elderly have reduced immune responses to many vaccines compared to young adults, attributed to immune senescence [7,13,33]. For example, it is recommended that an increased dose of influenza vaccine be used in individuals older than 60 years of age in an attempt to overcome immune senescence observed after vaccination. Studies of this phenomenon suggest that the deficit in elderly responses is due, at least in part, to defects in generating de novo responses to influenza vaccines containing new strain antigens, which is virtually always in the context of previous influenza infections/vaccinations [13,34,35]. Seasonal influenza vaccination in the elderly predominately activates memory B cells established in earlier infections/vaccinations [13,34], resulting in antibodies secreted by these memory B cells that are variably less protective against new influenza strains [35].

Most humans have been infected with RSV by two years of age but may be reinfected multiple times throughout life [1]. Different antigenic variants as the cause of repeated RSV infections have not been clearly documented. Some studies have shown that re-infections may be due to the same strain of RSV as the previous infection. Others are due to different RSV genotypes (reviewed in [14]). Re-infections with RSV may also be due, in part, to defects in the induction of effective anti-RSV memory responses upon the initial infection [1,36,37]. It is also possible that there is minimal durability of the antibodies induced in prior infections. Thus, the challenge for RSV vaccines is to identify more clearly what is responsible for re-infections and to formulate vaccines and protocols that will overcome these defects and induce protection in the context of non-protective immune responses due to prior infections.

Immune senescence in CRs has been documented by Guichelaar, et al. [12] and Boukhvalova, et al. [33], who showed that the induction of anti-RSV IgG, neutralizing antibody titers, and protection from RSV challenge was significantly lower in elderly CR compared to young CR. In these studies, however, the animals were RSV naïve, that is, they were not previously infected with RSV. The study reported here was accomplished in animals previously infected with RSV to mimic the human population. Our previous studies have demonstrated that a single injection of our novel vaccine candidates, virus-like particles, in young RSV-experienced (primed) adult cotton rats can activate memory established by a previous RSV infection and/or generate new protective responses resulting in dramatic increases in total anti-pre-F IgG, neutralizing antibodies, and protection from RSV challenge [15,16]. To determine if VLP immunization of RSV-experienced elderly animals also activated protective memory to RSV antigens, we compared properties of immune responses after VLP immunization of RSV-primed elderly versus young animals to assess the influence of age on activation of memory established by prior RSV infections. We addressed this question in several ways using two different protocols. Both approaches resulted in the same conclusion, that a single immunization of RSV-primed elderly animals or young animals resulted in the same levels of anti-pre-F IgG, anti-G IgG, neutralizing antibody titers, and protection from RSV challenge. Thus, VLPs were equally capable of activating protective memory in young and elderly animals.

However, by day 252 (36 weeks after an RSV prime), anti-pre-F IgG and anti-G IgG titers in animals receiving one immunization at an early age (Group 1 in protocol 2, Figure 3) declined with time to levels much lower than those in animals immunized at old age (Group 2). Surprisingly this decline was not reflected in a significant drop in NAb titers in group 1 animals (Figure 5 and Figure 6). The NAb titers in group 1 and group 2 at 256 days after RSV prime were very similar. These results suggest that immunization of young adults will provide significant protection from RSV infections as they age. Indeed, our results confirm this prediction as shown in Figure 7. Identification of the properties of the antibodies, such as specificities, isotype, Fc domain, glycosylation, and avidity, induced by immunizations in young vs. elderly populations and remaining by day 252 should provide further insight into why NAb titers and protection from challenge remain high in the face of declining levels of total anti-pre-F and anti-G IgG.

We extended these studies by defining the results of immunizing animals twice, once at a young age and once at old age with the goal of determining if two immunizations would improve protective responses. While a second immunization did increase levels of total anti-pre-F and anti-G IgG, the levels did not reach those after a single immunization in older animals, particularly in males. Furthermore, the second immunization did not significantly increase the NAb titers, and the second dose did not improve protection from the RSV challenge.

One important outcome of the study was to measure vaccine protection in primed, VLP-vaccinated animals by measuring viral mRNA transcription in the lung (Figure 7). Since only RSV-naïve animals generate viable viruses upon RSV challenge, to measure protection, primed animals need a different approach. We previously demonstrated that viral mRNA transcription is detectable in RSV-primed animals re-infected with RSV [32]. In addition, we showed this replication is associated with an increase in lung pathology. We found that animals, RSV-primed and immunized with VLPs at any point in life, were able to control the production of viral transcript significantly better than nonimmunized, RSV-primed animals. To our knowledge, this is the first report showing a correlate of protection from viral replication of a vaccine in the elderly using RSV-primed animals.

Lung pathology in animals has been described after RSV challenge of FI-RSV immunized animals (for example [38,39]). In animal models, it has also been shown that declining antibody titers after other vaccine candidate immunizations are associated with lung pathologies upon RSV challenge [40,41]. Thus, the safety of VLP immunization of the elderly was a concern. Group 1 animals in experimental design 2 immunized once at a young age, did experience significant declines in anti-pre-F and anti-G IgG titers. Indeed, these group 1 animals did show slightly increased levels, but not statistically significant, of pathology compared to groups 2 and 3, which had no decline in antibody levels at the time of the RSV challenge. However, the instances where lung pathology was previously reported were in RSV naïve animals immunized with either FI-RSV Lot 100 vaccine [42] or soluble RSV protein [41], or young animals with maternally transferred antibodies specific for RSV [43], and in the context of low antibody titers followed by RSV challenge [41]. In our studies, the animals were previously infected with RSV prior to immunization, a protocol which has resulted previously in suppression of subsequent lung pathology upon RSV challenge [44,45]. The results indicate that immunization of the elderly at either a young or old age with our VLP vaccine is safe.

Our studies confirm numerous prior observations that females respond with higher levels of immunity than males [31]. In measures of total IgG levels and NAb titers, we found that females virtually always had higher titers than males although in some instances the differences were not always statistically significant. However, the repeated findings of these differences emphasize the importance of including both sexes in studies of vaccine efficacy in order to obtain a more complete picture of responses in the entire population.

In conclusion, these studies demonstrate, in the cotton rat animal model, that prior RSV infection can induce protective memory and that our VLP vaccine candidates can activate that memory in elderly animals to provide enhanced protective levels of neutralizing antibodies and protection from RSV challenge. Furthermore, immunization in young animals provides will provide protection for the elderly without further immunization. However, a single immunization in old age also provides protection at least as good as a single immunization in the young or immunization at both a young age as well as an old age. In either case, immunization in the elderly, either as a primary or secondary immunization, results in reduced expression of RSV transcripts in the lung, and slightly less lung pathology upon RSV challenge than that seen after immunization only in young adults.

The results of this study support the idea that immunization of the young or elderly, with the appropriate RSV vaccine, is an excellent approach for the protection of this population. The results also support vaccination of younger adults for protection from RSV disease later in life. As suggested by Del Giudice et al. [46], vaccination during adulthood should be considered part of “health insurance” for protection from RSV disease during old age.

## Data Availability

Data are contained within the article or Supplementary Material.

## Supplementary Materials

The following supporting information can be downloaded at: https://www.mdpi.com/article/10.3390/v15061305/s1, Figure S1: Anti-pre-fusion F IgG titers in individual animals in Protocol Two; Figure S2: Photomicrographs of peribronchiolitis and perivasculitis in lung sections of RSV-challenged cotton rats; Figure S3: Photomicrographs of alveolitis in lung sections of RSV-challenged cotton rats.
