# Supplementary material for: Development of Respiratory Syncytial Virus Vaccine Candidates for the Elderly
Source: Viruses. 2023 May 31;15(6):1305. doi: 10.3390/v15061305 (PMC10304043; doi:10.3390/v15061305)
Supplement: Supplementary file 1 [file viruses-15-01305-s001.zip › viruses-2414504-supplementary.pdf]

Blanco et al.,
Supplemental Figures

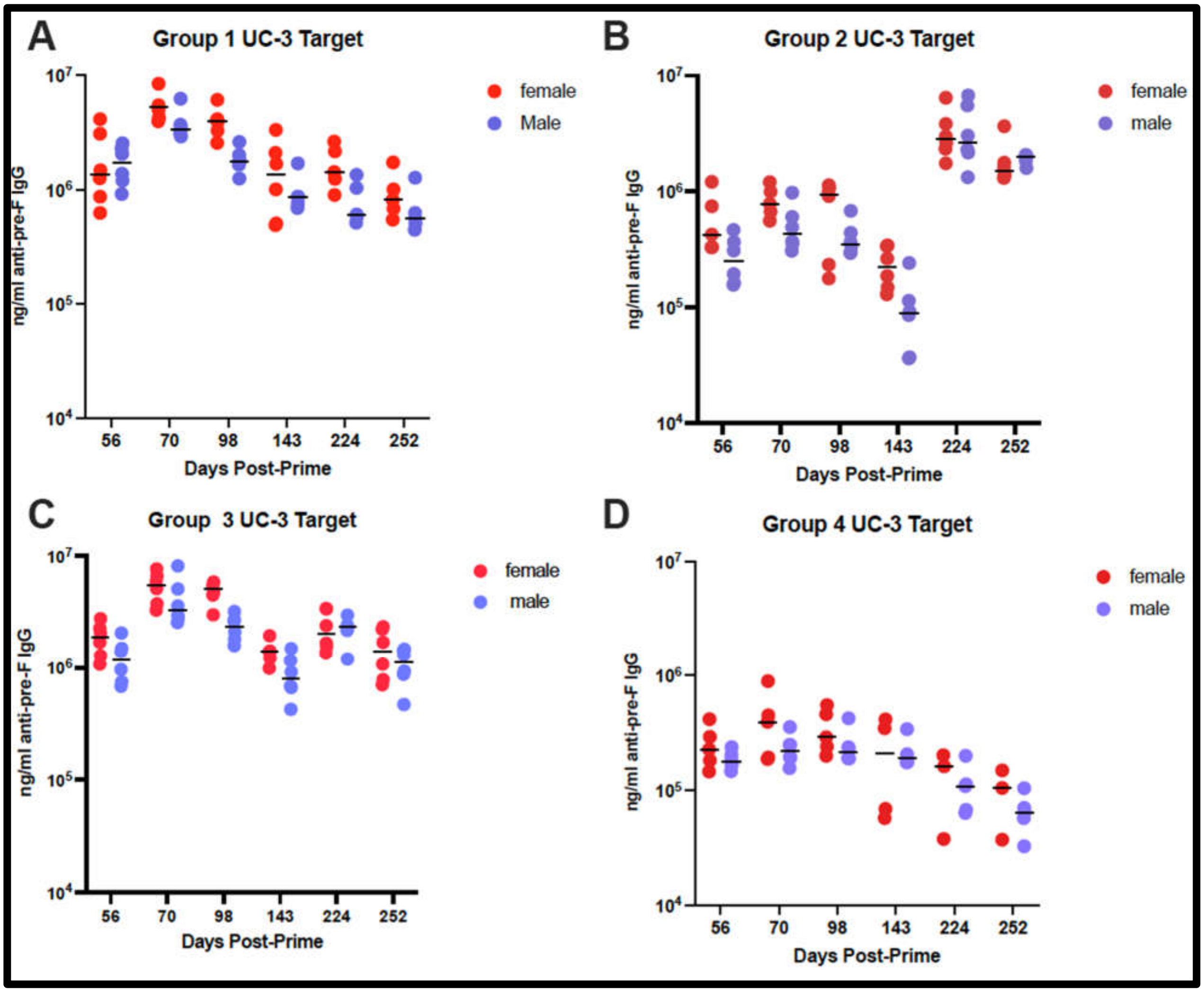

Legend to Supplemental Figure S1: Anti-pre-fusion F IgG titers in individual animals in protocol 2

The sera from individual females (red) and males (blue) in each group at each time point were analyzed separately. The ng/ml of anti-pre-F IgG in each serum were measured by ELISA with soluble UC-3 F protein as target (panels A-D). The averages of the results from sera of individual animals at each time point are indicated by a black line.

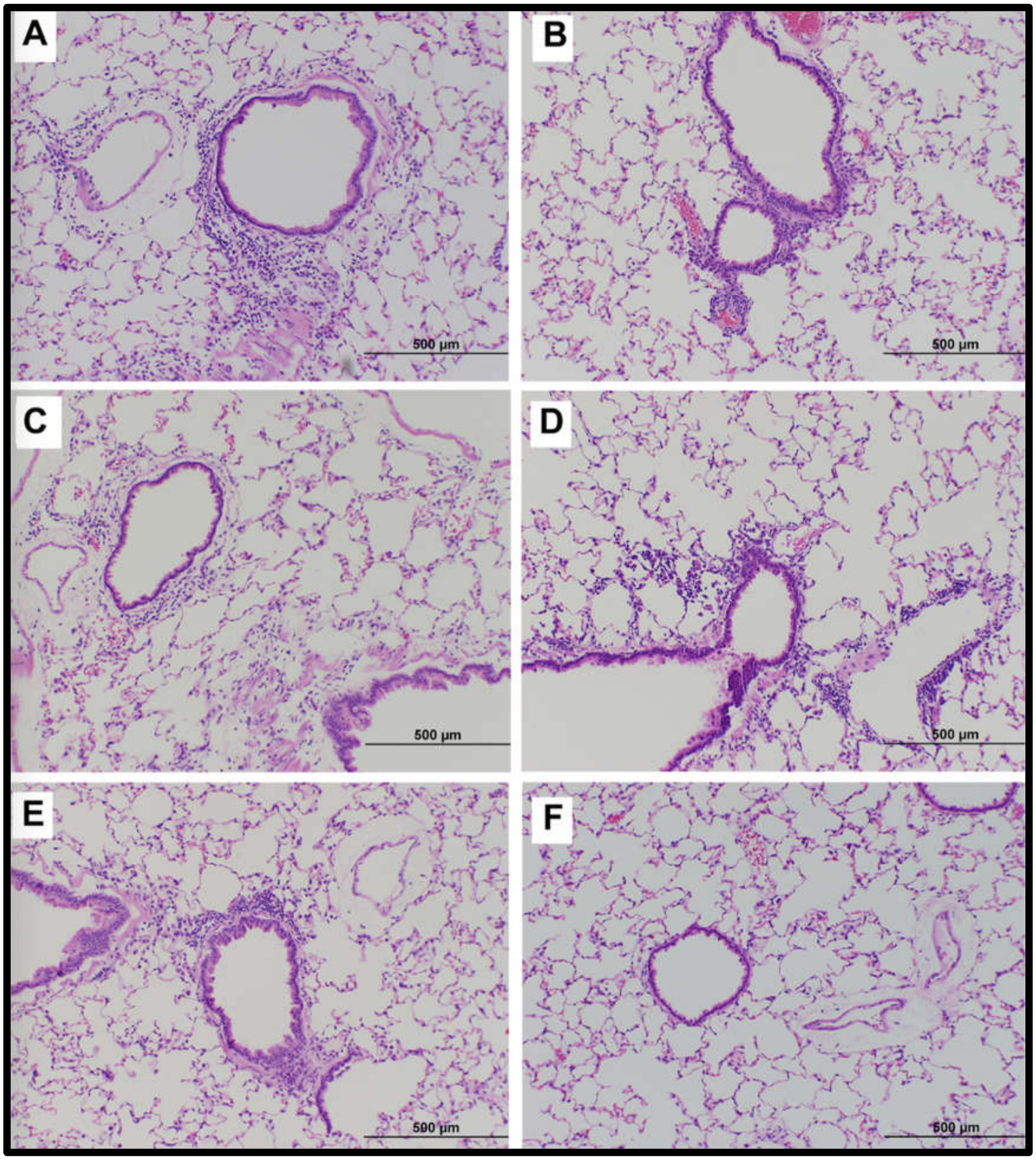

Legend to Supplementary Figure S2: Photomicrograph of peri bronchiolitis and perivasculitis in vaccinated animals in protocol 2 sacrificed 4 days after RSV challenge. A: VLP-Mock, group 1; B: Mock-VLP, group 2; C: VLP-VLP, Group 3; D: Mock-Mock, group 4; E: no primed, group 5; F: uninfected, group 6. H&E stain, x 100. The key features of peri bronchiolitis and perivasculitis are cell infiltration (lymphocytic and neutrophils) around the periphery of the bronchiole and blood vessels, respectively.

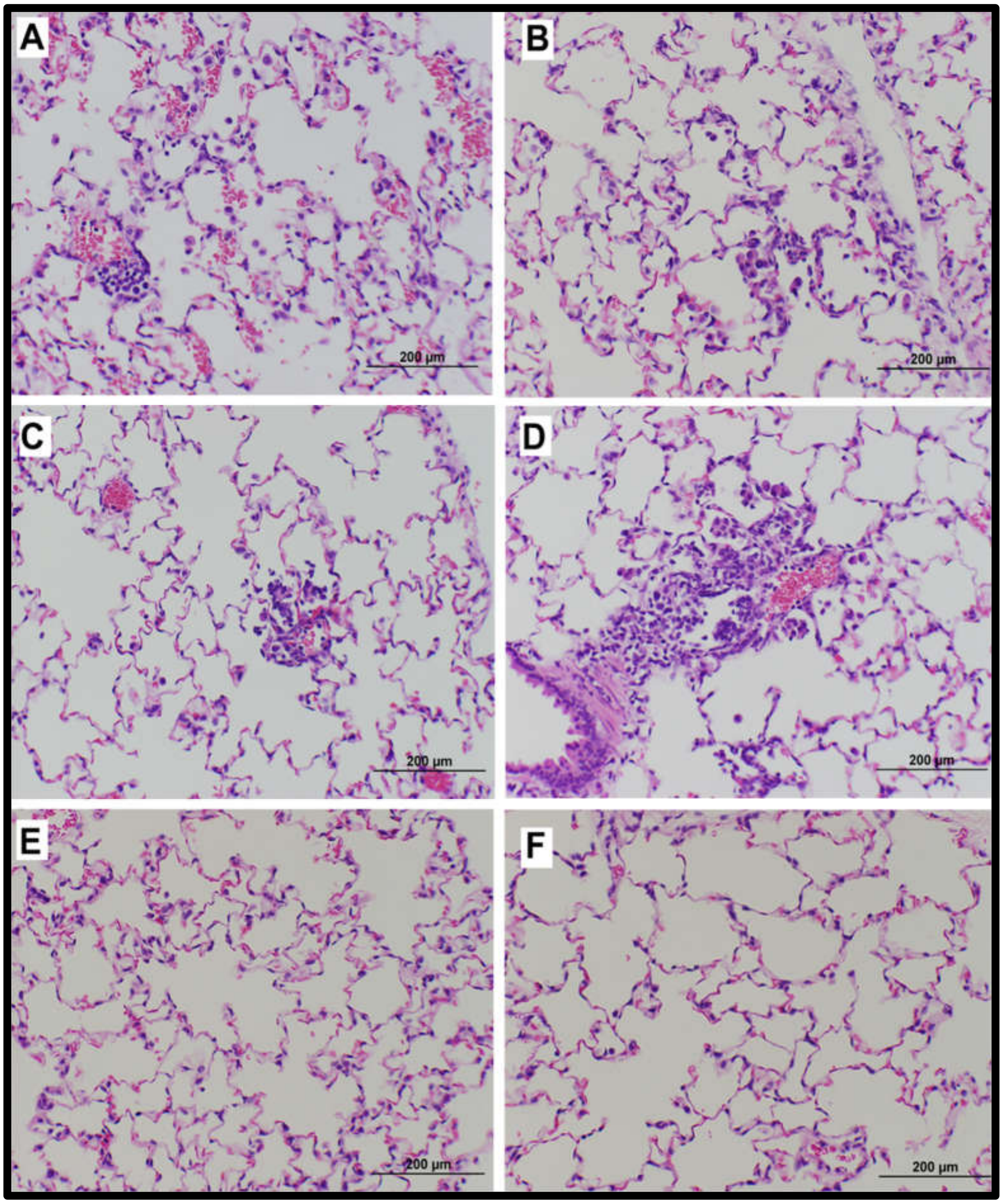

Legend to Supplementary Figure S3: Photomicrograph of alveolitis in vaccinated animals in protocol 2 sacrificed 4 days after RSV challenge. A: VLP-Mock, group 1; B: Mock-VLP, group 2; C: VLP-VLP, Group 3; D: Mock-Mock, group 4; E: no primed, group 5; F: uninfected, group 6. H&E stain, x 200. The key feature of alveolitis is inflammatory cells in alveolar spaces.
